# Elucidating the Mass Transportation Behavior of Gas Diffusion Layers via a H_2_ Limiting Current Test

**DOI:** 10.3390/ma16165670

**Published:** 2023-08-17

**Authors:** Min Wang, Wei Zhao, Shuhan Kong, Juntao Chen, Yunfei Li, Mengqi Liu, Mingbo Wu, Guanxiong Wang

**Affiliations:** 1 College of New Energy, China University of Petroleum (East China), Qingdao 266580, China; minwang@upc.edu.cn (M.W.); s22030109@s.upc.edu.cn (W.Z.); chjtbill@163.com (J.C.); 2015050107@s.upc.edu.cn (Y.L.); 2115050105@s.upc.edu.cn (M.L.); 2 College of Life Sciences, Nanjing Agricultural University, Nanjing 210095, China; 9211310305@stu.njau.edu.cn; 3 Shenzhen Academy of Aerospace Technology, Shenzhen 518057, China

**Keywords:** proton exchange membrane fuel cells, gas diffusion layer, mass transport resistance, limiting current, hydrogen probe

## Abstract

The gas diffusion layer (GDL), as a key component of proton exchange membrane fuel cells (PEMFCs), plays a crucial role in PEMFC’s polarization performance, particularly in mass transport properties at high current densities. To elucidate the correlation between GDLs’ structure and their mass transport properties, a limiting current test with the H_2_ molecular probe was established and employed to investigate three representative GDLs with and without the microporous layer (MPL). By varying humidity and back pressure, the mass transport resistance of three GDLs was measured in an operating fuel cell, and an elaborate analysis of H_2_ transport was conducted. The results showed that the transport resistance (R_DM_) of GDLs was affected by the thickness and pore size distribution of the macroporous substrate (MPS) and the MPL. In the process of gas transport, the smaller pore size and thicker MPL increase the force of gas on the pore wall, resulting in an increase in transmission resistance. Through further calculation and analysis, the total transport resistance can be divided into pressure-related resistance (R_P_) and pressure-independent resistance (R_NP_). R_P_ mainly originates from the transport resistance in both MPLs and the substrate layers of GDLs, exhibiting a linear relationship to the pressure; R_NP_ mainly originates from the transport resistance in the MPLs. 29BC with thick MPL shows the largest R_NP_, and T060 without MPL shows the R_NP_ = 0. This methodology enables in situ measurements of mass transport resistances for gas diffusion media, which can be easily applied for developing and deploying PEMFCs.

## 1. Introduction

Proton exchange membrane fuel cells (PEMFCs), as highly efficient energy conversion devices, directly convert the chemical energy from fuel and oxidant into electrical energy based on electrochemical principles [1,2,3,4]. However, in space-restricted applications such as automobiles or aviation, the total size of the power system should not be too large, and the weight should be as light as possible. As a balance, the fuel cell often operates at a current density higher than 1 A/cm^2^. In this high current density region, an increase in current density results in greater consumption of gas reactants, leading to a higher mass transport resistance. It significantly affects the overall performance of the fuel cells [5,6]. Thus, a thorough investigation into the effective mass transport properties of reactants/products in fuel cells operating under high current density operating conditions is of great importance.

The gas diffusion layer (GDL) is an important component of the membrane electrode assembly (MEA) which is located between the flow field and the catalyst layer (CL), supporting the CL and collecting currents, and providing multiple channels for gas, electrons, and water in the electrode reaction [7,8,9]. It mainly determines the transport property of gas reactants, removes liquid water, and affects the mass transfer of the fuel cell [10]. The performance of the PEMFC is highly influenced by transport resistance, especially in the high-current-density region. Therefore, it is of great importance to study the impact of GDL’s structures on fuel cell transport properties. Recently, researchers have studied the PEMFC performance effect of MPL porosity and pore size distribution. Chen et al. showed that micropores with pore sizes ranging from 0.5 to 15 μm, exhibited superior water management and gas transport properties within the GDL, benefiting the PEMFC’s overall performance. Chun et al. [11] successfully tuned the pore size distribution of the MPL by setting different drying conditions during the coating. The micropores showed better water removal effects, and the macropores could provide better gas transport. Proper combination and distribution of pores with different scales can achieve the best PEMFC performance. Zhan et al. [12] studied the effect of GDL porosity variation on liquid water flux, and the increase in MPL porosity decreased the saturation of liquid water in the GDL, which promoted the removal of water, thus enhancing the gas transport of GDL to the CL. The thickness of MPL also affects the mass transfer performance of PEMFC [13,14]. A too-thin MPL and a too-small pore size will hinder gas transport. On the other hand, when the MPL is too thick, the diffusion path becomes too long, resulting in greater resistance. Lin et al. [15] prepared MPL with different thicknesses to test the performance of PEMFCs, which varied with the adjustment of the thickness of the MPLs. The results showed that the MPL thickness reached the best performance at 30 μm, and the pore size distribution at this thickness was also the most favorable for gas transport. The work does not specifically discuss the effect of thickness on mass transport. The interface between the MPL and the CL also plays an important role in the total mass transport resistance [16,17]. When the surface roughness of MPL is large, the incomplete contact between layers can easily lead to the formation of gaps on the interface, increase the ohmic impedance, and also easily cause internal water to hinder gas transport [18,19]. Deng et al. [20] utilized three-dimensional (3D) imaging technology to investigate morphological variations in the GDL caused by different assembly pressures, subsequently establishing a simulation model for analysis. The results indicated that GDL compression led to increased surface roughness and decreased porosity, both of which contributed to a higher mass transport resistance in the PEMFCs. These studies revealed how the structural design and changes of GDLs affect water management, thereby indirectly affecting transport resistance. There are few studies to verify the effect of GDL structures by testing gas transport resistance.

Current studies typically employ an oxygen-limiting current method to measure gas mass transport resistance [21,22]. The reaction between O_2_ and H_2_ on the catalyst surface produces water and heat, resulting in inconsistent relative humidity and testing conditions with the setup conditions. This inconsistency might introduce local gas transport resistance, thereby affecting the accuracy of test results. In this study, a limiting current method with a H_2_ molecular probe to investigate the mass transport resistance of GDLs was deployed [23]. Thin platinum black layers deposited at the membrane interface served as electrochemical sensors, performing hydrogen oxidation for H_2_ probe gas molecules passing through GDLs. Compared to existing techniques, this H_2_-limiting current measurement avoids the generation of water or heat, resulting in reduced measurement disturbances and more reliable data. Moreover, the principles of this testing method can be extended to mass transport resistance testing in electrochemical hydrogen compressors (EHCs). EHC is a device that utilizes electrochemical principles to compress hydrogen gas to a higher pressure [24,25]. In an EHC, there is a certain mass transport resistance in the MEA involving the H_2_, H^+^, and H_2_O transfer [26]. When the mass transport resistance increases, more energy is needed to compress hydrogen to the target pressure, reducing the efficiency of the compressor. The hydrogen transport resistance directly determines the compression capacity and transport efficiency of hydrogen, which plays an important role in improving the performance and stability of EHCs [27,28,29]. Therefore, it is necessary and important to develop a facile and reliable technique to investigate the H_2_ transport resistances for EHCs and this H_2_ probe method provides a new approach.

To avoid the effect of water which is produced at the cathode of PEMFC on the mass transport behavior, we developed a limiting current method using a H_2_ molecular probe to investigate the GDL’s mass transport resistances. In this study, the MEAs containing different commercial GDLs were assembled for testing, and the mass transport resistance affected by GDL was extracted through calculation and analysis. Scanning electron microscopy (SEM), 3D profilometer, and mercury intrusion porosimetry (MIP) were conducted to investigate the structural properties of the GDLs. The correlation between the structure of GDL and their mass transport properties was further elucidated. This H_2_ limiting current methodology and research findings provide in-depth guidance for the mass transfer enhancement in energy conversion devices such as PEMFCs and EHCs.

## 2. Experimental Section

### 2.1. Materials

The Nafion^®^ 211 membrane (Dupont, Wilmington, DE, USA) was employed as the PEM. Commercial GDLs were Toray 060 (with 5% waterproofing), F91 (Freudenberg, Weinheim, Germany), and SGL29BC (Sigracet, Wiesbaden, Germany). The platinum catalysts were platinum black and TEC10E50E, TKK. 5 wt% Nafion^®^ solution (Dupont DE2020) was used as ionomer.

### 2.2. MEA Fabrication and Cell Assembly

The platinum black (PtB) electrode layer is referred to as the working electrode (WE) on the anode in this work, its thickness is about 2–3 μm, and the platinum load is 0.8 mg_Pt_/cm^2^. The ink of the PtB electrode layer was composed of water and n-propanol in the ratio of 4:3, the catalyst mass concentration was about 4 mg_Pt_/mL_ink_. The PtB electrode layer of the anode does not contain ionomers to prevent the adsorption of sulfate side chain groups on the PtB electrode’s surface, which could lead to Pt poisoning. A Pt catalyst supported on high surface area carbon (Pt/C) was used for the cathode, and the platinum load of 0.2 mg_Pt_/cm^2^. The Pt/C electrode layer was prepared Pt/C catalyst mixed with 5 wt% Nafion^®^ solution. The ratio of water to n-propanol in the ink solvent was 4:3, and the I/C ratio was 0.9.

**Figure 1:**
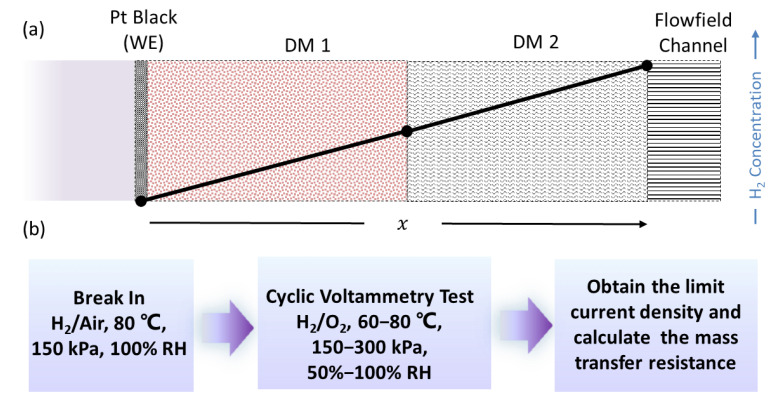
(**a**) Schematic diagram of H_2_ limiting current measurement; (**b**) Flow chart for testing protocols of H_2_ mass transport resistance tests via a limiting current method.

The specific structure of the MEA in this work is shown in Figure 1a. The cathode side adopts the conventional PEMFC cathode assembly method and the anode CL is PtB as WE. The GDLs on the anode side are the primary subject of mass transport characterization. For the test set up, a 14-serpentine flow field fixture was employed, with an MEA active area of 5 cm^2^ [30]. The flow field plate and end plate were securely assembled by tightening the fixture diagonally using octagonal screws, with a torque of 5 N·m. The compression ratios of Toray 060, F91, and SGL29BC were 20%, 18%, and 25%, respectively.

### 2.3. Limiting Current Tests

The schematic diagram of the H_2_-limiting current measurement and the testing protocol is illustrated in Figure 1b. Initially, the MEA was conditioned in H_2_/Air with an RH of 100% at 80 °C and a back pressure of 150 kPa. Subsequently, cyclic voltammetry (CV) testing was performed by introducing a H_2_/N_2_ mixed gas with a H_2_ concentration of 5% to the anode and cathode. The test parameters were set as follows: a cell temperature of 80 °C, back pressure ranging from 150 to 300 kPa, cell’s relative humidity (RH) ranging from 50% to 100%, a flow rate of 2000 and 5000 sccm in the cathode and anode, respectively. The desired limiting current density value was determined using the CV method [8]. The platform current with a voltage higher than 0.4 V was recorded as the limiting current value. The mass transport resistance of the GDL was then calculated based on the acquired limiting current density value, and the detailed calculation process has been described in our previous work [31].

### 2.4. Experimental Design to Test the Mass Transport Resistance of GDL

At least two components in PEMFC components contribute to the total gas transport resistance, including the gas flow channel (CH) and the gas diffusion medium (DM), which typically contains MPL. Since these components are arranged in series, the total mass transport resistance is the cumulative sum of their individual resistances, which is shown in Equation (1).

(1) $$R_{\mathrm{Tot}}=R_{\mathrm{CH}}+R_{\mathrm{DM}}+R_{\mathrm{MPL}}+R_{\mathrm{Other}}$$

In the equation, R_Other_ represents the mass transport resistance of all other gases in the hydrogen fuel cell, R_CH_ represents the mass transport resistance of the gas flow channel, R_DM_ represents the gas mass transport resistance of the DM (excluding the MPL), and R_MPL_ represents the gas mass transport resistance of MPL.

It should be noted that DM is composed of a substrate layer and a very thin MPL. Due to differences in porosity, the mass transport mechanisms of the substrate layer and MPL are completely different. Therefore, MPL is represented separately, where the mass transport resistance of MPL is R_MPL_. In the substrate layer of DM, the molecular size of the gases is much smaller than the pore diameter, so the gas molecules will not collide with the walls when passing through the pores and can move freely [32]. At this time, gas transport resistance is dominated by intermolecular diffusion [33]. On the other hand, in the MPL, due to its very low porosity, the interaction between reactant molecules and pore walls is more frequent and intense than molecular collisions, making Knudsen diffusion the main diffusion mode [34].

When N DMs are assembled in series (N ≥ 2), the total mass transport resistance can be expressed as:

(2) $$R_{\mathrm{Tot}}=R_{\mathrm{CH}}+{\mathrm{NR}}_{\mathrm{DM}}+{\mathrm{NR}}_{\mathrm{MPL}}+R_{\mathrm{Other}}$$

Therefore, if two DMs are assembled in the cell and the total mass transport resistance calculated is R_Tot2_, subtracting the total mass transport resistance measured by assembling one DM, which is R_Tot2_ − R_Tot1_, it is the mass transport resistance R_DM_ of one DM. In this process, both R_CH_ and R_Other_ have been removed as background data.

In order to further verify the feasibility of this method and confirm that the background data has been completely eliminated, another cell is designed with a target GDL, consisting of three DMs. The total mass transport resistance is R_Tot3_. If the above assumptions hold true, according to the linear relationship hypothesis, R_DM_ will satisfy:

(3) $$R_{\mathrm{DM}}=R_{\mathrm{Tot}2}-R_{\mathrm{Tot}1}=R_{\mathrm{Tot}3}-R_{\mathrm{Tot}2}=\frac{1}{2}\left(R_{\mathrm{Tot}3}-R_{\mathrm{Tot}1}\right)$$

Via calculations, it is possible to determine the mass transport resistance of the target layer with various DMs under different gas pressure and relative humidity conditions.

### 2.5. Structure Characterizations

The cross-sections of three types of GDLs were characterized using SEM (JEOL 7000 F FE-SEM), and GDL cross-sections were obtained utilizing the liquid nitrogen freeze-fracture method [35]. The 3D profilometer (Keyence model VHX5000, Osaka, Japan) was employed to study the GDL’s surface morphology. The MIP (Micromeritics AutoPore IV 9600, Norcross, GA, USA) was employed to characterize the size distribution of macro and mesopores in the three GDL structures.

## 3. Results and Discussions

### 3.1. The Mass Transport Resistance of T060

**Figure 2:**
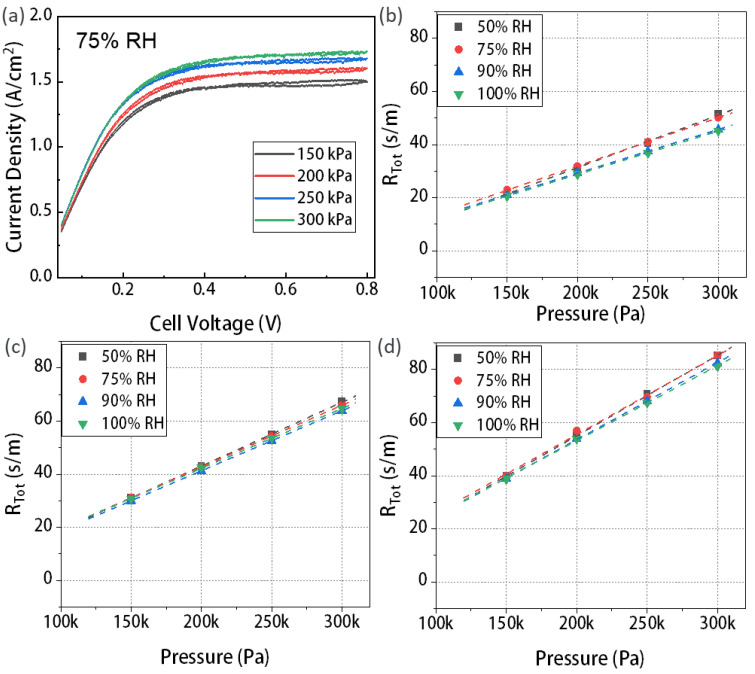
(**a**) The CV curves of T060 tested under four cell pressures at a RH of 75%; total transport resistance of T060 under variable RHs and pressures with different GDL layers: (**b**) one layer, (**c**) two layers, and (**d**) three layers.

The limit current density of T060 tested under different pressures is plotted in Figure 2a and Figure S1. The impact of pressure on the limited current density is more pronounced under high humidity conditions. This is attributed to the larger proportion of water vapor in the total gas mixture at higher RH [36]. With increasing pressure, the change in reactant concentration is greater in high-humidity conditions compared to low-humidity conditions. When maintaining a constant pressure, the difference in limit current density caused by different RHs is not significant. This is due to the fact that when RH increased from 75% to 100%, the concentration of reactants in the gas flow channel did not undergo substantial changes, only decreasing by approximately 5–10%.

According to the formula presented by Greszler et al. [37], as shown in Figure S2,

(4) $$R_{\mathrm{Tot}}\left(P\right)=\mathrm{mP}+b$$

The total resistance (R_Tot_) is linearly related to the gas pressure (P), where m and b are constants that can be experimentally determined.

Furthermore, considering the influence of different diffusion mechanisms on gas pressure, it can be observed that when the total pressure (P) remains constant, the binary diffusion coefficient for molecular diffusion is inversely proportional to P. On the other hand, the diffusion coefficient for Knudsen diffusion in the microporous structure remains independent of pressure. Consequently, the total transport resistance of gas in a fuel cell can be divided into pressure-dependent resistance R_P_, and pressure-independent resistance R_NP_

(5) $$R_{\mathrm{Tot}}=R_{P}+R_{\mathrm{NP}}$$

According to the transport mechanism, it is known that the intercept part of the straight line of the R_Tot_–P relationship diagram is the transport resistance independent of the gas pressure P, that is, the transport resistance R_NP_ in MPL; the part above the intercept is the transport resistance related to the gas pressure P, which is the transport resistance R_P_ in the substrate layer. The total mass transport resistance of T060 was calculated with one, two, and three GDL layers under different pressures and RHs. Figure 2b shows the mass transport resistance measured for a single layer of T060.

We observe a phenomenon that the intercept from the curve to the *y*-axis is 0. Since T060 only contains the substrate layer as its cross-section image shown in Figure S3, the pressure-independent resistance resulting from Knudsen diffusion in the MPL is 0. The same results are observed for the two- and three-layer configurations. In addition, when the RH is kept constant, the total mass transport resistance increases with increasing pressure. This is because as the pressure rises, the concentration of reactants in the gas channel increases. It leads to more intense molecular collisions during gas transportation, thus increasing the mass transport resistance [38]. The experimental data further confirm the linear relationship between pressure and total mass transport resistance in Figure S2. Further effects in different RHs were analyzed. Under equal pressure conditions, the mass transport resistance in low humidity conditions is slightly higher than that in high humidity conditions. This may be attributed to the PEM not reaching sufficient humidity in low-humidity conditions, despite the high concentration of reactants in the channel, thereby affecting gas transport and reactions [39]. Furthermore, by comparing the trend of GDL transport resistance with pressure for one, two, and three layers, it is observed that as the number of layers increases, the slope of the straight line also increases. This indicates an increasing pressure-related resistance caused by molecular diffusion in the GDL, which can be attributed to the greater number of pores. Specifically, at an RH of 75% and a pressure of 250 kPa, the total mass transport resistance of the cell with one layer, two layers, and three layers was determined to be 41 s/m, 54.3 s/m, and 69.6 s/m, respectively from Figure 2b–d.

**Figure 3:**
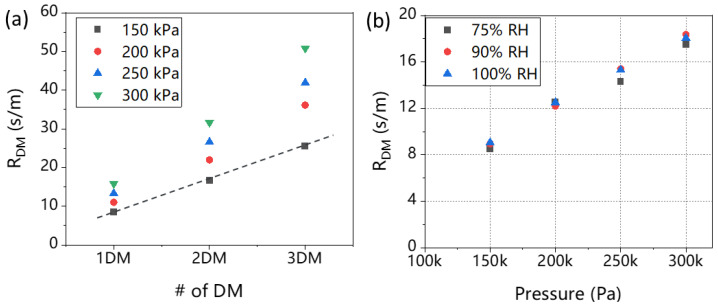
(**a**) The R_DM_ of T060 with different DMs under variable pressure at a RH of 75%. (**b**) The R_DM_ of GDL under variable RHs and pressure for T060 with one layer.

By performing calculations on the total mass transport resistance of T060 with different layers under varying pressures and RHs, we further derived the mass transport resistance caused by the GDL. Figure 3a shows the equidistant sequence relationship of mass transport resistance for one, two, and three layers of GDL at an RH of 75% which confirmed the reliability of Formula (3). The transport resistance of the GDL increases as the number of layers increases. Specifically, at 150 kPa, the change in mass transport resistance increased from 8.5 s/m for one layer of GDL to 25.5 s/m for three layers of GDL. Similarly, at 200 kPa, the mass transport resistance increased from 15.8 s/m for one layer to 50.8 s/m for three layers. This can be attributed to the greater total thickness in the GDL as the number of layers increased with the increase in the thickness, the gas transport paths become longer, increasing the tortuosity [40]. On the other hand, the collision frequency between the gas and the hole through the GDL increases, resulting in an increase in mass transport resistance [41].

Figure 3b depicts the changes in the R_DM_ for a single layer of GDL under different pressures and RHs. Under the same pressure, the impact of RHs is insignificant on the R_DM_ of the GDL. This since when the RH was low, the humidity difference was large, and water vapor would transfer faster from the cathode side to the anode side, resulting in a higher mass transfer resistance. However, when the RH reached 75%, the saturated water vapor pressure was already high enough, and the transfer of water vapor was no longer significantly restricted. In this case, the change in mass transport resistance will not be significant. In addition, the intercept of the curve at the *y*-axis was 0, which further validated the conclusion that the pressure-independent resistance caused by the Knudsen diffusion was indeed 0 due to the absence of the MPL in T060 [42].

### 3.2. Structure Characterizations

**Figure 4:**
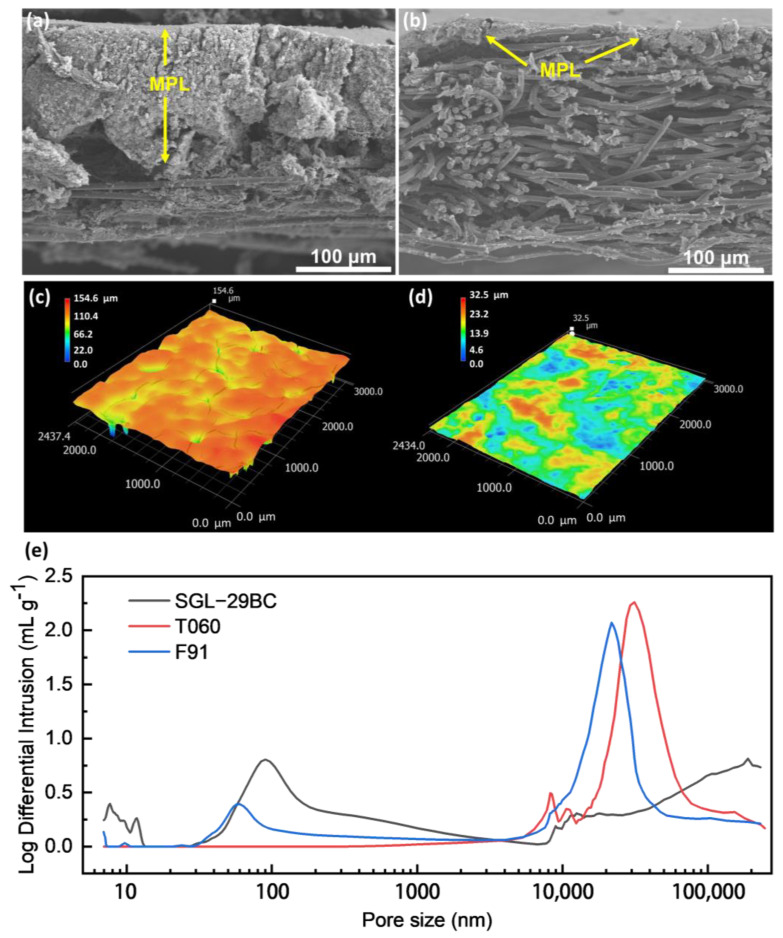
SEM image of cross-section (**a**) SGL-29BC (**b**) F91; surface AFM diagram (**c**) SGL-29BC (**d**) F91; (**e**) pore size distribution data obtained from MIP method measurement.

Figure 4 shows the differences in the GDL structures and pore size configurations using three characterization methods, which explored the influence of GDL structure on the change of mass transport resistance. Figure 4a,b show a cross-sectional view of SGL29BC (29BC) and F91. All studied GDLs have distinctive fine/coarse structures made of carbon nanoparticles and carbon fibers, respectively. From the SEM images, it is observed that the GDL thickness of F91 and 29BC was similar. However, the thickness ratio between fine and coarse regions differs between the studied GDLs. F91 has an MPL/substrate ratio of 22/208 μm/μm, while 29BC has a larger ratio of 100/130 μm/μm. Visual inspection of SEM micrographs additionally reveals a deeper penetration of MPL into carbon paper substrate for 29BC compared to F91. Figure S3 shows a cross-sectional view of T060, which only had the substrate composed of carbon fibers. Such structural differences may significantly influence the pore-size distribution in the GDLs.

Figure 4c,d show more details on the surface structure of studied GDLs. The 3D profilometer is utilized to analyze the surface morphology, structural information, and surface roughness of the different GDL samples. The presence of cracks and a rougher surface in 29BC results in a difference of approximately 150 μm between the highest and lowest points. Conversely, the surface of F91 appears smoother, with only a 30 μm difference between the highest and lowest points without an apparent crack [43]. This difference in surface roughness may impact the mass transport resistance of the GDLs.

To study the effect of pore-size distribution in more detail, we investigated samples using MIP. Figure 4e shows the pore-size distribution of studied GDLs. The whole pore-size spectrum can be divided into three parts: micro- (<100 nm), meso- (0.1–2 μm), and macro-pore (>2 μm) regions. T060 excluding MPL does not appear a distinctive peak in the micro-porous range. 29BC and F91 which contain MPL, demonstrate a distinctive peak in the micro-porous range. The formation of pores with a size of ∼100 nm is typical for systems based on carbon black particles as previously reported [44]. Notably, 29BC has the largest peak in this region which suggests the highest loading of carbon black particles. Moreover, a significant penetration of carbon black particles into the substrate, revealed by the SEM for 29BC, has an impact on the pore-size distribution in the macro-porous region [32]. Regarding the macro-porous region, all studied GDLs have pores of 10–40 μm which is typical for the carbon paper in the GDL substrate. These differences of pore size distribution may be attributed to the characteristics of different support materials. Further, we discussed the structural effects of 29BC and F91 GDLs on mass transport properties.

### 3.3. The Mass Transport Resistance of SGL-29BC and F91

**Figure 5:**
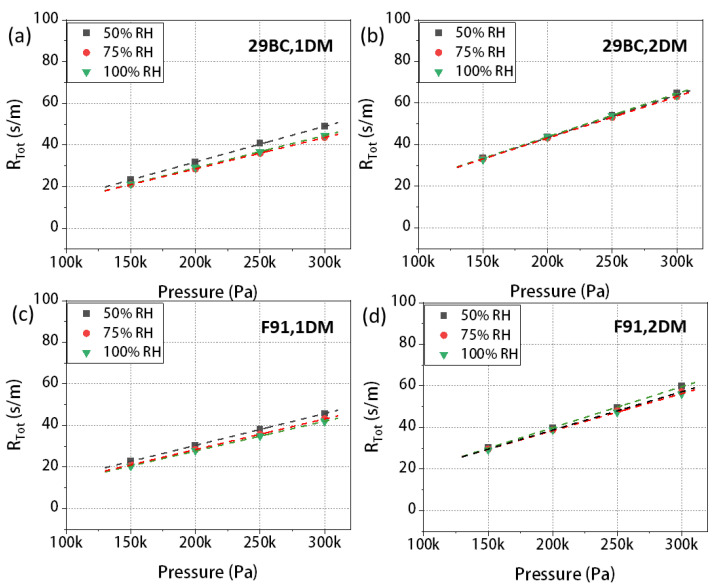
The total transport resistance of SGL 29BC under variable RHs and pressures, (**a**) one layer, (**b**) two layers; total transport resistance of F91 under variable RHs and pressures, (**c**) one layer, (**d**) two layers.

Figure 5 shows the total transport resistance of 29BC and F91 under variable RHs and pressures. The pristine data points of one and two layers of 29BC were plotted, and the total mass transport resistance values were calculated for each RH as shown in Figure 5a,b. The phenomenon we observed in T060 also occurred here which was caused by saturated water vapor pressure: under the same pressure, as RHs increased from 50% to 100%, the mass transport resistance decreased [43]. Additionally, since both types of GDLs contain a substrate layer and an MPL, the intercept of the curve from the *y*-axis (representing the pressure-independent resistance) is greater than 0. The intercept of 29BC is slightly larger than that of F91, which was possibly due to the difference in MPL’s thickness and pore structure.

**Figure 6:**
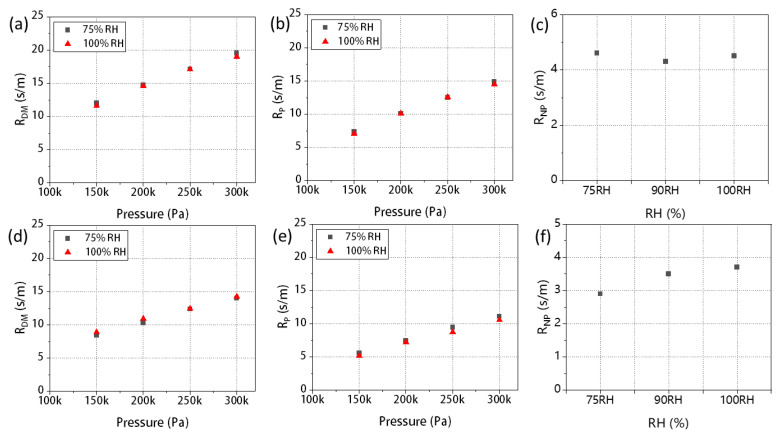
The R_DM_ of GDL under variable RHs and pressures (**a**) SGL-29BC (**d**) F91; pressure-related mass transport resistance R_P_ (**b**) SGL-29BC (**e**) F91 in GDL under variable RH and pressure conditions; pressure-independent mass transport resistance R_NP_ (**c**) SGL-29BC (**f**) F91 in GDL at 75% and 100% RH.

By analyzing the original data, we were able to isolate the components related to the R_DM_ caused by the GDL in the fuel cell, which as shown in Figure 6a,d. We observe that the two GDLs have similar total mass transport resistance, but the R_DM_ has a quite difference. The R_DM_ of 29BC is slightly greater than F91. This difference is attributed to their distinct MPL thickness and pore size distribution. The MPL of 29BC is thicker, the pore size distribution is mainly in the microporous area with a denser distribution of carbon black particles. Thus, the gas transport path is more tortuous, resulting in a higher gas transport resistance [44].

Subsequently, we further divided R_DM_ into two parts, R_P_ and R_NP_. Figure 6b,e show the R_P_ of the two GDLs. We discovered a notable characteristic of the two GDL types: the mass transport resistance R_P_ is proportional to gas pressure, which can be explained by molecular diffusion theory [45]. When the channel diameter is much larger than the average molecular free path (i.e., molecular average free path/2r ≤ 1/100, where r is the average channel radius) [28], collisions between molecules mainly occur during their movement. The proportion of collisions between molecules and pore walls is very small, and an increase in pressure intensifies intermolecular collisions, leading to an increase in mass transport resistance [46]. Under the same pressure, the R_P_ of 29BC is higher than that of F91. Specifically, at 100% RH and a pressure of 250 kPa, the R_P_ of 29BC was 12.59 s/m, while F91 had an R_P_ of 8.7 s/m. This disparity can be attributed to the structure of 29BC which has a dense distribution of micropores, resulting in gas diffusion obstruction. The data for the R_NP_, which is pressure-independent, is presented in Figure 6c,f. Comparing the mass transport resistance under different RH conditions, we found that RH had a minimal effect on R_NP_. When the RH increased from 75% to 100%, the R_NP_ of 29BC only increased by 0.1 s/m. In addition, 29BC with thicker MPL also exhibits a higher R_NP_.

### 3.4. Comparison of Mass Transfer Resistance in GDLs

**Figure 7:**
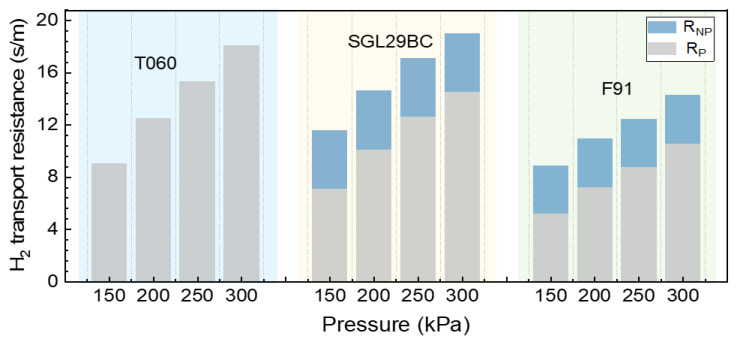
Comparison diagram of total hydrogen transport resistance in different GDLs: T060, SGL29BC, F91 under 75% RH.

Figure 7 summarizes the mass transport resistance of three GDLs with different pore structures based on the above analysis. The R_DM_ of the three GDLs has the same trend, which increases with the increase in pressure. The R_DM_ of T060 is slightly lower than that of SGL 29BC, and F91 exhibits the lowest mass transfer resistance. T060 does not contain microporous layers, while F91 and SGL 29BC have an MPL. It should be noted that the R_DM_ is not only relevant to the structure of the macroporous substrate, but also impacted by the microporous layer’s thickness and pore size distribution. The R_DM_ of T060 may come from the MPS. The R_DM_ of SGL 29BC and F91 originates from both MPS and the MPL. To further understand the effect of the MPL, we divided the R_DM_ into R_P_ and R_NP_. T060′s transport resistance is mainly caused by molecular diffusion, and its R_NP_ is 0. Both 29BC and F91 contain MPL, so the R_NP_ ≠ 0. Furthermore, the MPL of 29BC is thicker, has more micropores and a more tortuous gas transport path. As a result, the increased number and denser arrangement of micropores in this GDL extended the diffusion distance of hydrogen molecules, leading to an elevation in mass transport resistance and consequently an increase in R_NP_ [47].

## 4. Conclusions

In this work, we focused on studying the H_2_ transport properties of three commercial GDLs. By changing the layer number of GDLs in MEAs and combining it with the limiting current method, the H_2_ transport resistance in different GDLs was successfully measured in different RHs and pressure. We conclude:

(1) The influence of humidity on the transport resistance of H_2_ in the GDL is relatively small and the impact of back pressure on H_2_ transport resistance is significant and positively correlated. An increase in pressure resulted in a higher concentration of reactants in the gas flow channel. This makes collisions between gas molecules more violent, thereby linearly increasing the total mass transport resistance.
(2) The R_DM_ of GDLs is mainly dominated by porosity, pore size distribution, and thickness of both MPSs and MPLs. In the process of gas transport, the smaller pore size and porosity increase the force of gas on the pore wall. This will reduce the effective transport efficiency of hydrogen gas, resulting in an increase in transport resistance. On the other hand, with the increase in thickness, the gas transport paths become longer, increasing the tortuosity.

Through further analysis, the total transport resistance was divided into pressure-related resistance R_P_ and pressure -resistance R_NP_, and the main influence sources of the two kinds of resistance were explored in detail:

(1) R_P_ mainly comes from the MPL and substrate layers in GDL and is linearly related to pressure. In the substrate layer of DM, gas transport resistance is dominated by intermolecular diffusion, which is greatly affected by pressure. R_NP_ mainly comes from MPL in GDL. Due to the extremely low porosity, Knudsen diffusion becomes the main diffusion mode and is less affected by pressure.
(2) T060 without a MPL does not show R_NP_; R_P_ and R_NP_ are successfully separated for SGL 29BC and F91. The R_NP_ of 29BC with a thicker MPL is higher. The increased number and denser arrangement of micropores in this GDL extended the diffusion distance of hydrogen molecules. It leads to an elevation in mass transport resistance and consequently an increase in R_NP_.

## Data Availability

Not applicable.

## Supplementary Materials

The following supporting information can be downloaded at: https://www.mdpi.com/article/10.3390/ma16165670/s1, Figure S1: The limit current density curve of T060 layer tested under four cell pressures at different RHs (a) 50%; (b) 100%; Figure S2: Linear relationship curve between cell pressure and total transport resistance; Figure S3: The SEM image T060 cross section.
