# Supplementary material for: Elucidating the Mass Transportation Behavior of Gas Diffusion Layers via a H2 Limiting Current Test
Source: Materials (Basel). 2023 Aug 17;16(16):5670. doi: 10.3390/ma16165670 (PMC10456699; doi:10.3390/ma16165670)
Supplement: Supplementary file 1 [file materials-16-05670-s001.zip › materials-2555499-supplementary.pdf]

Supporting Information

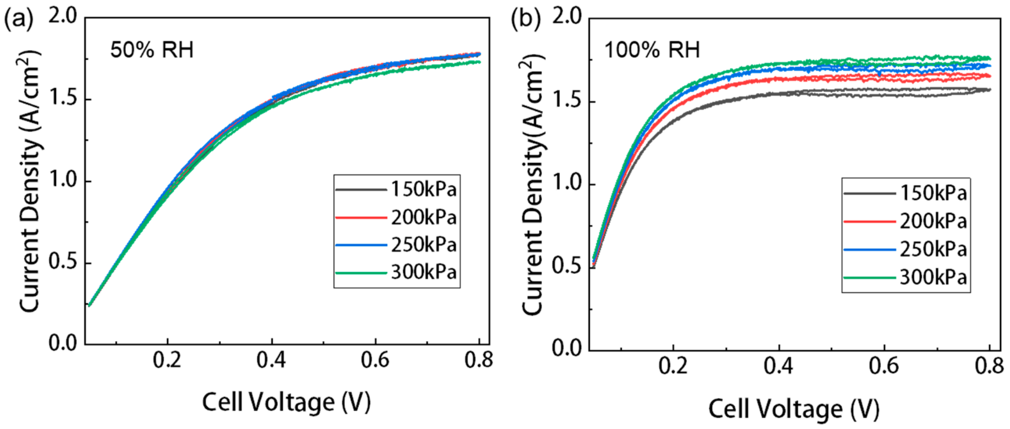

Figure S1. The limit current density curve of T060 layer tested under four cell pressures at different RHs (a) 50%; (b) 100%.

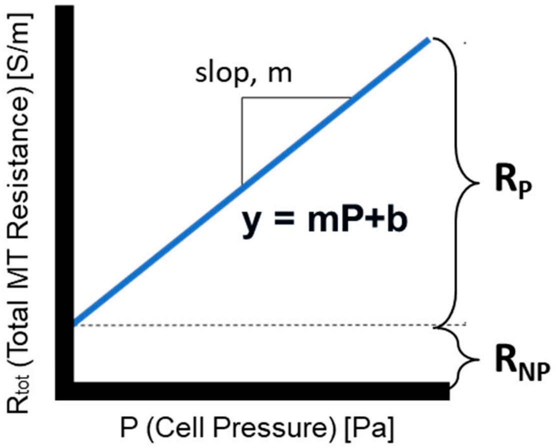

Figure S2. Linear relationship curve between cell pressure and total transport resistance.

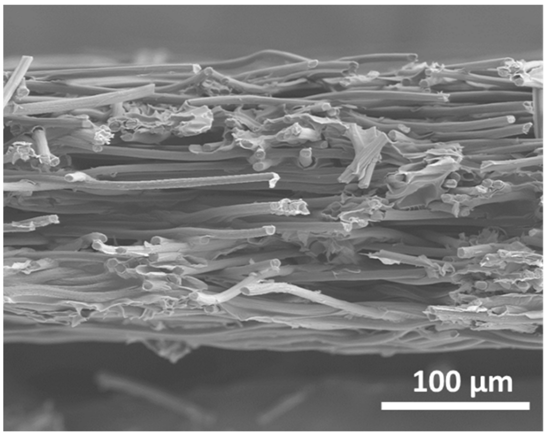

Figure S3. The SEM image T060 cross section.
